# Technical evaluation of non-coplanar lattice radiotherapy: achieving directional VPDR uniformity with a 5-mm leaf width multi-leaf collimator

**DOI:** 10.3389/fonc.2026.1807931

**Published:** 2026-05-22

**Authors:** Young Kyu Lee, Chan-beom Park, Yunji Seol, Jin-Ho Song, Kyu Hye Choi, Ji Hyun Hong, Wonjoong Cheon, Young Nam Kang, Byung-Ock Choi

**Affiliations:** 1 Department of Radiation Oncology, Seoul St. Mary’s Hospital, College of Medicine, The Catholic University of Korea, Seoul, Republic of Korea; 2 Department of Medical Sciences, College of Medicine, The Catholic University of Korea, Seoul, Republic of Korea

**Keywords:** bulky tumor treatment, directional dose uniformity, lattice radiotherapy, medical linear accelerator, non-coplanar beam delivery, phantom study, spatially fractionated radiation therapy, valley-to-peak dose ratio

## Abstract

**Purpose:**

Spatially fractionated radiation therapy (SFRT) creates alternating high-dose peaks and low-dose valleys for treating bulky tumors, with optimal biological effects associated with sufficient spatial dose modulation across the tumor volume. Coplanar lattice radiotherapy (LRT) demonstrated significant directional imbalance: superior-inferior VPDR was 2.7-fold lower (14.5%) than anterior-posterior and lateral directions (approximately 40%), with transverse directions exceeding the dosimetric range commonly reported in prior LRT studies. This study systematically evaluated non-coplanar LRT to quantify directional VPDR uniformity improvements using the Millennium 120 multi-leaf collimator.

**Methods:**

A 3×3×3 lattice structure with 27 vertices was implemented in a cylindrical phantom across 20 geometric configurations: vertex diameters 0.5-2.0 cm and separations 1.0-5.0 cm. Four non-coplanar arcs utilized couch angles of 0°, 315°, 45°, and 90° with consistent 45°collimator angle, optimized using Eclipse HyperArc. VPDR was analyzed in three orthogonal directions, normal tissue dose characteristics were assessed, and treatment delivery efficiency was evaluated through monitor unit distribution patterns and modulation complexity scores.

**Results:**

Direct comparison with coplanar delivery demonstrated substantial improvements in directional uniformity. For representative 1.5 cm diameter with 2.0 cm separation, non-coplanar delivery maintained superior-inferior VPDR at 26.4 ± 5.3% while reducing excessive transverse direction values from approximately 40% (coplanar) to 27.6 ± 1.5% and 25.8 ± 3.5% (non-coplanar), bringing all directions within the dosimetric range commonly reported in prior LRT studies. Excessively high transverse VPDR values (38-44% in coplanar) were reduced, achieving directionally balanced VPDR values across all vertex diameters. Normal tissue intersection volumes with high-dose regions achieved zero at separations ≥2.0 cm for 1.0 cm diameter and ≥3.0 cm for 0.5 cm diameter. Monitor unit distribution remained balanced across couch angles.

**Conclusions:**

Non-coplanar delivery successfully brought all three directional VPDR values within the dosimetric range commonly reported in prior LRT studies at separations ≥2.0 cm across vertex diameters, addressing the excessive transverse direction VPDR (>40%) observed in coplanar delivery. This advancement enables directionally balanced SFRT dose distribution in all directions, critical for uniform spatial fractionation effectiveness. This systematic evaluation provides quantitative evidence and parameter selection guidance for clinical implementation using conventional medical linear accelerators.

## 1 Introduction

Spatially fractionated radiation therapy (SFRT) creates alternating high-dose regions (vertices) and low-dose regions (valleys) within the target volume, showing promising therapeutic outcomes particularly for bulky tumors (1–3). The biological mechanisms include bystander effects, vascular damage, immune response activation, and enhanced reoxygenation of hypoxic regions (4, 5). Optimal biological effects have been reported when the valley-to-peak dose ratio (VPDR), defined as the ratio of minimum valley dose to maximum peak dose, is minimized while maintaining adequate normal tissue protection (2, 6). Clinical implementations have reported VPDR values in the 20-30% range (6), representing technically achievable dosimetric values with commonly used delivery systems. However, it should be noted that a clinically validated optimal VPDR range has not yet been established, as clear correlations between specific VPDR values and clinical outcomes remain to be determined. The primary dosimetric concern addressed in this study is therefore not the absolute VPDR value, but rather the pronounced directional imbalance — a 2.7-fold difference between SI and transverse directions — observed in coplanar delivery.

Initially implemented using physical GRID blocks, SFRT faced limitations including dose calculation difficulties, limited flexibility in vertex configuration, and technical complexities in block fabrication and alignment (7, 8). The evolution of multi-leaf collimator (MLC) technology enabled lattice radiation therapy (LRT), offering dynamic beam shaping, precise dose control, and flexible vertex arrangement (9, 10). Modern medical linear accelerators equipped with high-resolution MLCs (such as the Millennium 120 MLC with 5 mm central leaf width), six-degree-of-freedom treatment couches enabling sub-millimeter positioning accuracy, and advanced treatment planning systems supporting inverse optimization, now enable sophisticated three-dimensional LRT delivery (11–14).

Lee et al. (2025) systematically evaluated coplanar LRT using the Millennium 120 MLC across vertex diameters ranging from 0.5 to 2.0 cm and edge-to-edge separations from 1.0 to 5.0 cm using a 3×3×3 lattice arrangement (15). Their coplanar approach utilized four complete arcs with different collimator angles while maintaining a fixed couch angle of 0°. The study demonstrated that clinically relevant VPDR values could be achieved with appropriate parameter selection. However, a critical limitation emerged: significant directional imbalance in VPDR distribution. For a representative configuration (1.5 cm vertex diameter, 2.0 cm separation), transverse directions (AP: 39.7 ± 1.2%, LAT: 40.3 ± 1.1%) substantially exceeded the dosimetric range commonly reported in prior LRT studies, while the superior-inferior direction (14.5 ± 0.8%) fell well below this range (15). This pattern persisted across all configurations, with superior-inferior VPDR consistently lowest regardless of vertex parameters.

The pronounced directional imbalance raises important considerations for SFRT optimization. LRT has demonstrated improved normal tissue sparing compared to conventional approaches, and the three-dimensional distribution of sublethal doses affects overall tissue damage and repair capacity (9, 10). Recent studies also demonstrate that SFRT can enhance immune-mediated tumor control through spatially modulated dose delivery, with the spatial pattern of dose heterogeneity potentially influencing immune activation (16, 17). Therefore, achieving balanced VPDR distribution across all three orthogonal directions represents an important dosimetric goal for optimal SFRT implementation, though the clinical significance of directional uniformity requires biological validation through dedicated studies (18).

The directional VPDR imbalance in coplanar delivery originates from fundamental beam geometry. When all beams rotate within a single axial plane, beam trajectories naturally traverse multiple vertices in transverse directions, elevating valley doses and resulting in VPDR values exceeding 30%. In contrast, the superior-inferior direction experiences more focused dose deposition with rapid fall-off between axially separated vertices, achieving lower VPDR values (6). Non-coplanar beam arrangements, combining couch rotation with gantry rotation, offer a potential solution by introducing beam paths with superior-inferior directional components. This geometric reconfiguration is expected to redistribute dose more uniformly across all three dimensions, increasing superior-inferior VPDR while simultaneously reducing excessive transverse VPDR below the 30% threshold (6).

Dosimetric and geometric characteristics of LRT have been systematically investigated in recent years using C-arm linac-based delivery systems. Prado et al. reported geometrical and dosimetrical parameters including VPDR values and vertex configurations from initial clinical experience with single-fraction LRT for bulky tumors (19). Kavanaugh et al. established planning design and dosimetric endpoints for a Phase I clinical trial of Lattice SBRT, providing comprehensive dosimetric characterization across a broad patient population (20). Additional studies have reported successful LRT implementation across various clinical settings (10, 21, 22). However, none of these investigations have specifically evaluated the directional uniformity of VPDR distribution across three orthogonal axes. The pronounced directional imbalance between SI and transverse VPDR values observed in coplanar delivery (15) represents a dosimetric characteristic that has not yet been systematically evaluated or resolved for non-coplanar delivery across comprehensive vertex parameter ranges using identical methodology. This knowledge gap creates practical challenges for clinical implementation, as clinicians and medical physicists lack quantitative evidence necessary for evidence-based decisions regarding delivery approach selection and parameter specification.

This study addresses these limitations through comprehensive technical evaluation of non-coplanar LRT using the Millennium 120 MLC system. Our investigation employs identical phantom setup, vertex configurations, and dosimetric analysis methodology as the Lee et al. coplanar study (15), enabling direct quantitative comparison. We systematically evaluated 20 geometric configurations (vertex diameter: 0.5-2.0 cm; separation: 1.0-5.0 cm) using a 3×3×3 lattice structure with 27 vertices. Four key aspects were analyzed: (1) directional VPDR distribution and degree of balance improvement compared to coplanar delivery, (2) normal tissue dose characteristics including mean dose and high-dose volume metrics, (3) treatment delivery efficiency through monitor unit requirements, and (4) plan complexity using modulation complexity scores. These findings aim to establish a quantitative framework for non-coplanar LRT implementation, providing direct comparison with coplanar delivery and enabling evidence-based decisions regarding delivery approach and parameter selection for clinical applications.

## 2 Materials and methods

### 2.1 Phantom configuration and three-dimensional lattice structure

**Figure 1:**
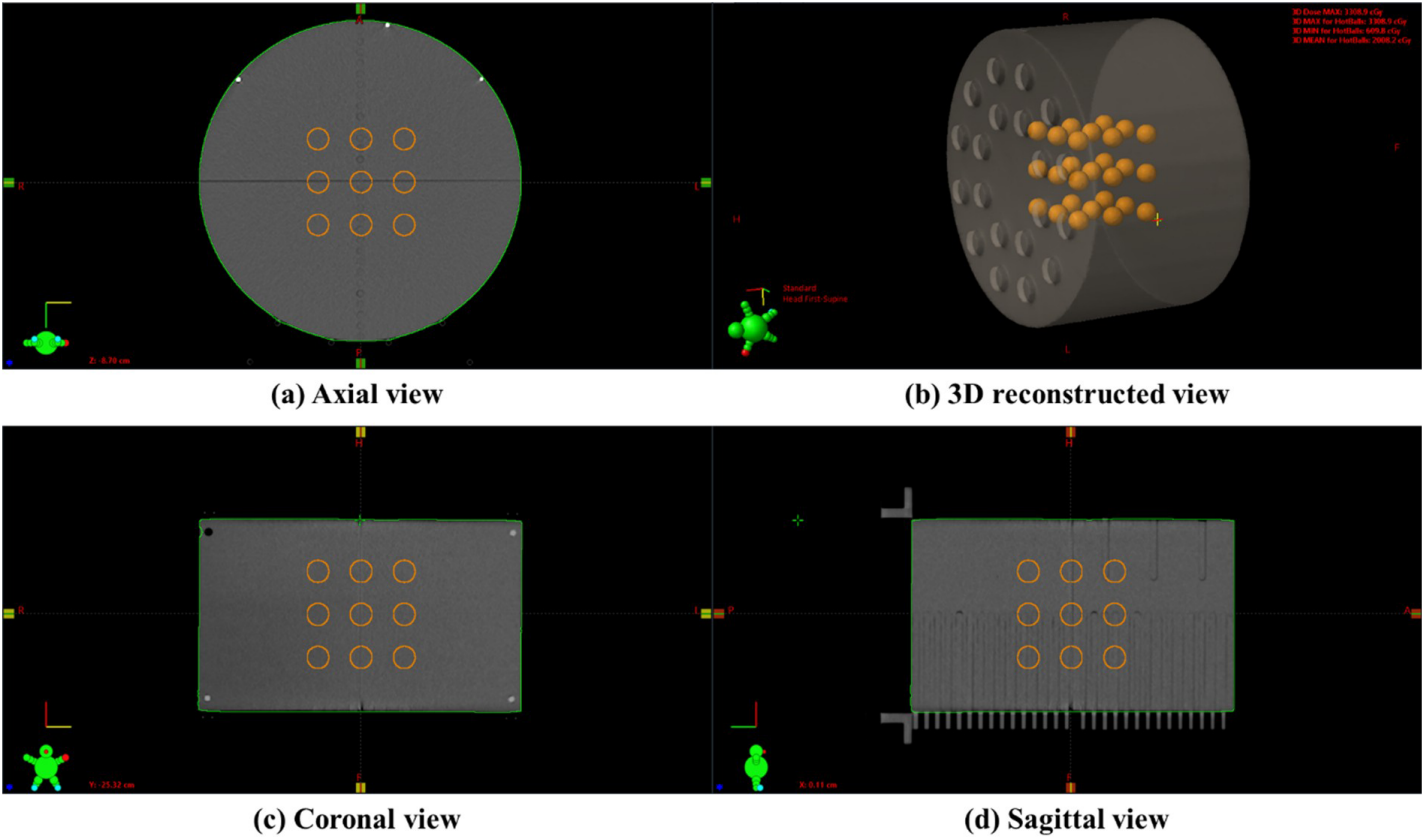
An example of a 3D lattice structure in the Virtual Water™ phantom: **(a)** axial view showing the cross-sectional arrangement of vertices, **(b)** 3D reconstructed view demonstrating the overall spatial distribution, **(c)** coronal view showing the superior-inferior vertex distribution, and **(d)** sagittal view illustrating the anterior-posterior vertex arrangement. This figure was reproduced from Lee et al. (15).

For the implementation and VPDR analysis of non-coplanar LRT using medical linear accelerators, as shown in **Figure 1**, we used a cylindrical Virtual Water™ phantom (Gammex RMI, Middleton, WI), which demonstrates the 3D lattice structure from (a) axial view, (b) 3D reconstructed view, (c) coronal view, and (d) sagittal view. The phantom has a diameter of 30 cm and a length of 18 cm. This phantom is equivalent to water in radiation absorption and scattering properties. CT images of the phantom were acquired using the SOMATOM go.Open Pro CT scanner (Siemens Healthineers, Germany) with a slice thickness of 1.0 mm.

Using in-house software, we created a 3×3×3 lattice structure within the phantom, consisting of twenty-seven high-dose vertices. The diameters of these vertices ranged from 0.5 cm to 2.0 cm in 0.5 cm increments. The separation, defined as the edge-to-edge distance between adjacent vertices, varied from 1.0 cm to 5.0 cm in 1.0 cm increments. This resulted in 20 distinct series, each with a unique combination of vertex diameter and separation. Radiation therapy plans were established for each of these series.

### 2.2 Treatment planning and non-coplanar beam configuration

Radiation treatment plans were optimized for delivery on a TrueBeam (Varian Medical Systems, Palo Alto, CA, USA) equipped with a Millennium 120 MLC (Varian Medical Systems, Palo Alto, CA, USA). The Eclipse version 16.0 treatment planning system (Varian Medical Systems, Palo Alto, USA) with HyperArc functionality was employed for radiation treatment planning. The Millennium 120 MLC features 60 leaf pairs with 5 mm central leaf width (inner 40 leaf pairs) and 10 mm outer leaf width (outer 20 leaf pairs). The prescribed dose was set to deliver 20.0 Gy to 50% of the vertex volume in a single fraction using 6 MV flattening filter-free (FFF) beams with a dose rate of 1400 MU/min. Non-coplanar beam delivery utilized four volumetric modulated arc therapy (VMAT) arcs with varying couch angles, as determined by the HyperArc optimization algorithm. All arcs maintained a consistent collimator angle of 45°. The couch-gantry angle combinations were: Arc 1 (couch 0°, gantry 180.1°-179.9°), Arc 2 (couch 315°, gantry 179.9°-0°), Arc 3 (couch 45°, gantry 0°-180.1°), and Arc 4 (couch 90°, gantry 180.1°-0°). The optimization was performed using the photon optimizer (PO) algorithm, and the final dose was calculated using the anisotropic analytical algorithm (AAA) (12) with a 1.0 mm dose calculation grid size.

**Figure 2:**
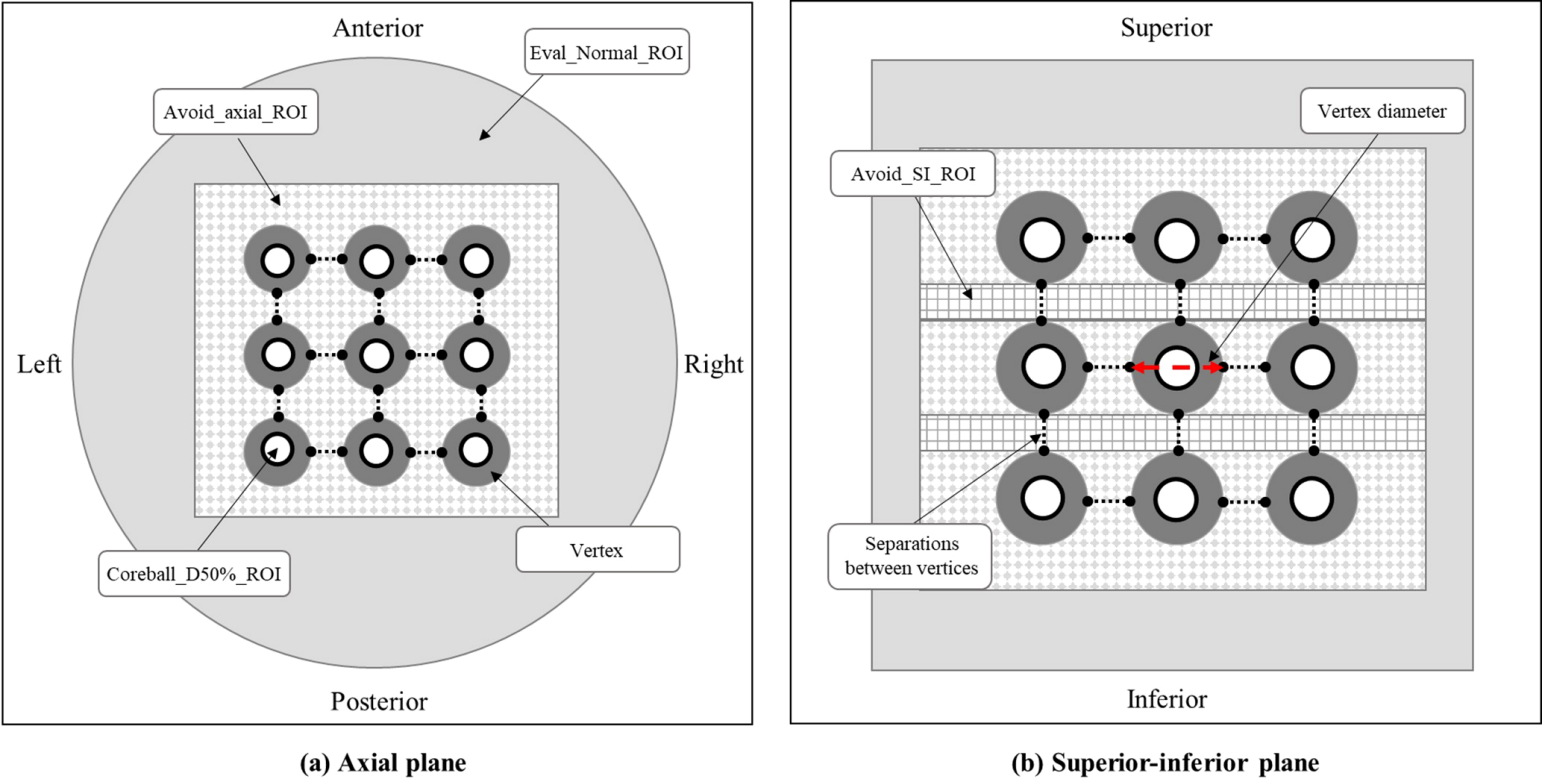
Vertices and regions of interest (ROI) configuration: **(a)** axial plane and **(b)** superior-inferior plane views showing the arrangement of vertices and various planning ROIs.

As shown in **Figure 2**, various regions of interest (ROIs) were established to achieve the planning objectives. The Coreball_D50%_ROI was defined to incorporate a spherical region with its diameter equal to half that of the vertex, positioned at the center of each vertex to ensure maximum dose delivery at the center point. A total of twenty-seven Coreball_D50%_ROIs were generated independently, enabling individual dose distribution control for each vertex. In the treatment planning optimization process, the minimum dose of Coreball_D50%_ROI was set to the prescription dose of 20.0 Gy. The optimization priority was adjusted to ensure that 50% of the vertex volume receives 50% of the prescription dose, thereby maintaining consistent and adequate dose delivery across all vertices. Through independent control of each vertex, we could ensure that the minimum dose met the prescription dose while maintaining dose uniformity between vertices.

Avoid_axial and Avoid_SI are two ROIs created to minimize the dose between the vertices. These ROIs are dynamically configured based on the diameter and separation of the vertices. Each ROI serves to reduce radiation dose in the surrounding area, excluding the high-dose region of the vertices themselves. The Avoid_axial ROI focuses on dose reduction in the axial plane, particularly in the X and Y-axis directions, while the Avoid_SI ROI emphasizes dose reduction in the superior-inferior direction along the Z-axis. Both avoid_ROIs are formed as rectangular prisms extending 1.0 cm from the outermost vertex ends, ensuring that their dimensions adapt according to variations in the diameter or separation of the vertices.

Eval_Normal_ROI was created to assess the dose to normal tissue. Eval_Normal_ROI is defined as the entire phantom volume excluding the avoid_ROIs. This configuration allows for the evaluation of dose to surrounding normal tissue, assuming the vertices are located within tumors.

**Figure 2** illustrates the configuration of vertices and ROIs in axial and SI planes for the radiation treatment plan. It shows the arrangement of high-dose vertices, Coreball_D50%_ROI, and avoidance structures (Avoid_axial_ROI and Avoid_SI_ROI). This procedure facilitates the optimization of dose delivery to the vertices while enabling the evaluation of dose distribution in surrounding normal tissue.

### 2.3 Valley-to-peak dose ratio analysis

**Figure 3:**
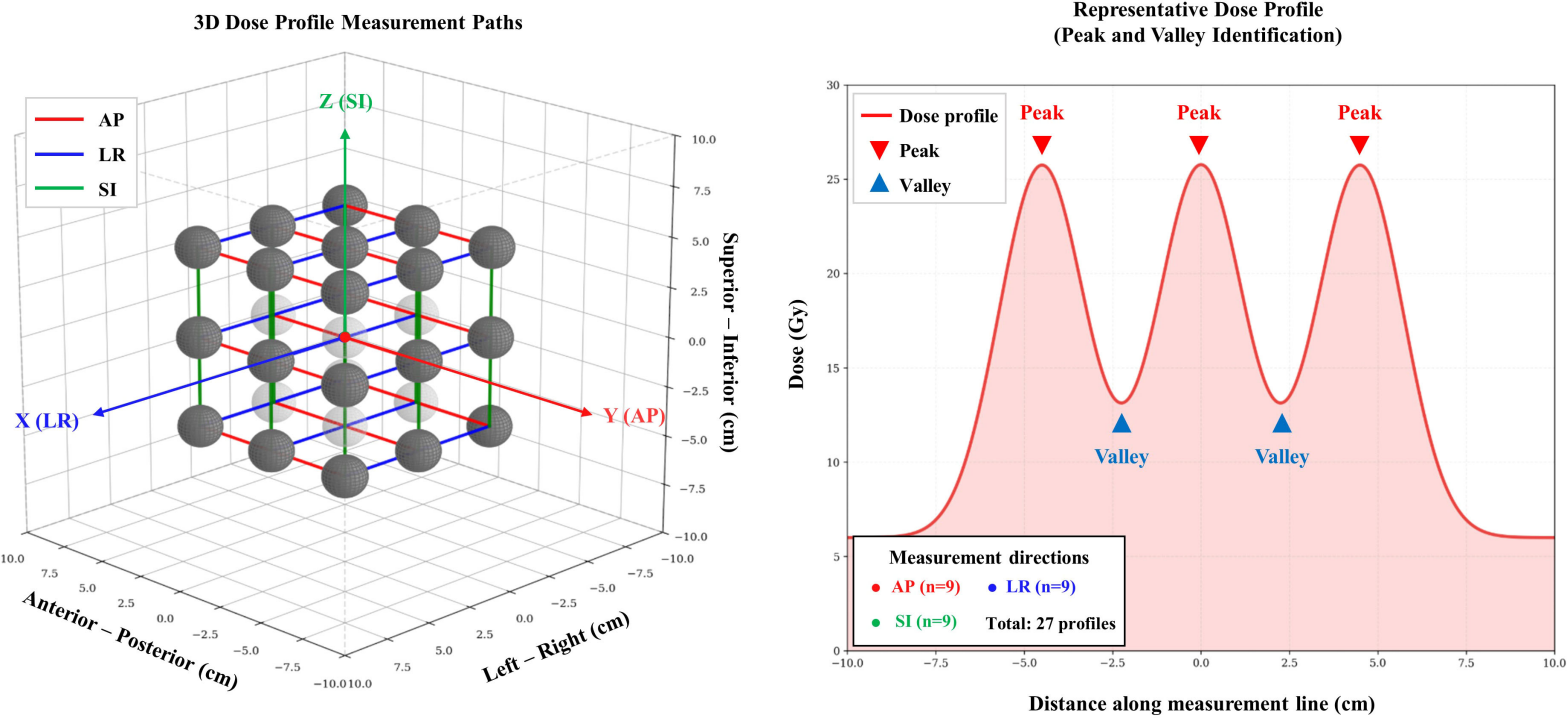
Method for acquiring dose profiles in three orthogonal directions and identification of peak and valley dose locations. Left panel shows the measurement paths along the anterior-posterior (n=9, red solid line), left-right (n=9, blue solid line), and superior-inferior (n=9, green solid line) directions. Right panel demonstrates a representative dose profile with peak and valley points.

VPDR values are used as a significant metric for evaluating the dose distribution characteristics and treatment effectiveness of SFRT. To calculate the VPDR, as shown in **Figure 3**, a total of 27 dose profiles were obtained in each plan from the anterior-posterior (AP), lateral (LAT), and superior-inferior (SI) directions, demonstrating the methodology for acquiring dose profiles and identifying peak and valley dose locations. To obtain dose profiles in each orthogonal direction, the maximum dose point (peak) within each vertex volume was individually identified using MATLAB (MathWorks, Natick, MA, USA). Subsequently, the minimum dose point (valley) within the region between two consecutive vertices was independently identified, where the inter-vertex region was defined as the spatial domain extending from the boundary of one vertex to the boundary of the adjacent vertex. This approach yielded an alternating sequence of peak and valley dose values (peak–valley–peak–valley) along each profile direction, without assuming geometric collinearity of the dose maxima across vertices. Since the identified peak and valley locations may not be perfectly collinear in three-dimensional space, inter-point distances along the profiles were normalized using MATLAB to generate uniformly spaced dose profiles, ensuring consistent and reproducible VPDR calculations across all 20 geometric configurations. This methodology was applied independently for each of the nine profiles in each orthogonal direction (AP, LAT, and SI), yielding nine VPDR values per direction per configuration. The VPDR is defined by the following formula (Equation 1):

(1) $$\text{VPDR}=\frac{D_{valley}}{D_{peak}}\;\times \;100$$

Where D_peak_ represents the maximum dose within each vertex volume individually identified using MATLAB, and D_valley_ represents the minimum dose within the region between two consecutive vertices (defined as the spatial domain extending from the boundary of one vertex to the boundary of the adjacent vertex), also identified using MATLAB. The alternating peak–valley–peak–valley sequence was extracted along each profile direction, with inter-point distances normalized to ensure uniform spacing. The mean and standard deviation of the VPDR obtained for each direction were calculated. To quantify directional uniformity, the coefficient of variation was calculated across the three orthogonal directions (AP, LAT, and SI) for each geometric configuration, defined as the ratio of the standard deviation to the mean expressed as a percentage. To statistically evaluate directional VPDR differences, pairwise comparisons between the three orthogonal directions (AP vs LAT, AP vs SI, and LAT vs SI) were performed using independent samples t-tests for each of the 20 geometric configurations. To account for multiple comparisons (3 pairwise comparisons × 20 configurations = 60 total comparisons), Bonferroni correction was applied, resulting in a corrected significance threshold of α = 0.05/60 = 0.0008. Statistical analyses were performed for both non-coplanar and coplanar delivery to quantitatively demonstrate the improvement in directional uniformity achieved by non-coplanar beam configuration.

### 2.4 Normal tissue dose, treatment delivery efficiency, and plan complexity analysis

Quantitative analyses were performed to evaluate the dosimetric performance of LRT plans. The mean dose within Eval_Normal_ROI was measured, and the volume intersecting the 50% prescription dose region (intersecting volume) was extracted to analyze the high dose delivered to normal tissues.

To evaluate the efficiency of the treatment plans, four non-coplanar arcs were utilized. The total monitor units (MU) for each plan were analyzed to assess the characteristics of each plan. Additionally, to quantify the contribution of each arc within an individual treatment plan, the MU ratio of each arc relative to the total MU was calculated. The modulation complexity score (MCS) was calculated to evaluate plan complexity (23), which considers leaf sequence variability and aperture area variability, expressed as a value between 0 and 1 (with 0 indicating highest complexity and 1 indicating lowest complexity).

### 2.5 Representative patient-based planning case

To evaluate the clinical applicability of the proposed non-coplanar LRT approach, a representative patient-based planning case was performed using a brain tumor patient CT obtained under institutional review board approval (IRB No. KC26RNSI0185). A spherical gross tumor volume of 173.8 cm³ was contoured, and non-coplanar LRT was planned with a vertex diameter of 1.0 cm and an edge-to-edge separation of 1.5 cm. A separation of 1.5 cm was selected as an intermediate value between the 1.0 cm and 2.0 cm separations evaluated in the phantom study, to accommodate the tumor volume while maintaining adequate vertex spacing. Due to the tumor volume constraints, 7 vertices were placed within the target volume. All other planning parameters, including beam configuration, couch angles, collimator angle, and dose prescription, were identical to those used in the phantom study.

## 3 Results

### 3.1 Directional valley-to-peak dose ratio distribution

**Figure 4:**
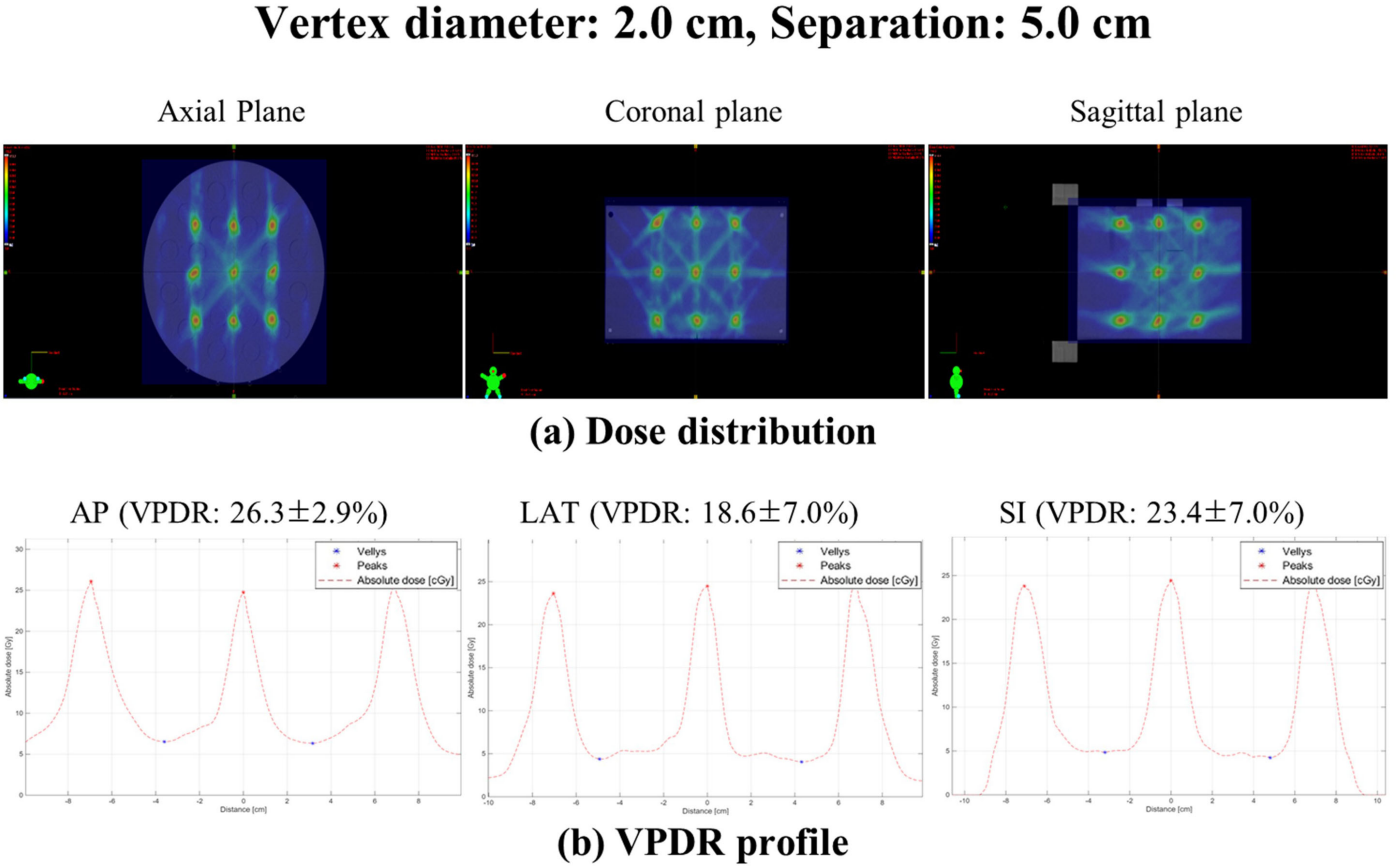
Representative dose distribution and VPDR profiles for vertex diameter 2.0 cm with 5.0 cm separation. **(a)** dose distribution in axial, coronal, and sagittal planes demonstrating the three-dimensional lattice structure. **(b)** VPDR profiles in AP (26.3 ± 2.9%), LAT (18.6 ± 7.0%), and SI (23.4 ± 7.0%) directions showing peak and valley dose patterns. Peak doses represent the maximum dose within each vertex volume and valley doses represent the minimum dose within the region between two consecutive vertices (defined as the spatial domain extending from the boundary of one vertex to the boundary of the adjacent vertex). Both values were identified using MATLAB with inter-point distance normalization.

The VPDR analysis for non-coplanar LRT configurations revealed systematic variations across vertex diameters (0.5-2.0 cm) and separations (1.0-5.0 cm), with distinctive directional characteristics compared to coplanar delivery. **Figure 4** illustrates representative dose distributions and VPDR profiles for the 2.0 cm diameter with 5.0 cm separation configuration. Comprehensive VPDR data across all 20 geometric configurations are presented in **Table 1**.

**Table 1:** Analysis of valley-to-peak dose ratio variations according to vertex geometry in non-coplanar lattice radiation therapy.

Valley-to-peak dose ratio (%)						
Diameter (cm)	Direction	Separation (cm)				
		1	2	3	4	5
**0.5**	AP	46.4 ± 2.9	27.8 ± 1.5	25.3 ± 1.9	22.1 ± 1.4	18.9 ± 1.8
	LAT	41.0 ± 4.6	25.3 ± 2.9	20.9 ± 3.2	19.1 ± 3.6	16.2 ± 3.4
	SI	41.9 ± 6.3	24.7 ± 4.0	22.0 ± 4.8	19.9 ± 3.8	17.5 ± 3.6
**1.0**	AP	36.6 ± 1.8	24.8 ± 0.5	23.1 ± 1.4	19.9 ± 1.9	16.3 ± 1.3
	LAT	32.9 ± 4.1	21.4 ± 3.0	19.4 ± 3.7	17.2 ± 3.7	13.3 ± 2.8
	SI	32.7 ± 5.5	20.2 ± 3.4	18.5 ± 3.0	18.7 ± 4.0	15.5 ± 3.0
**1.5**	AP	36.1 ± 0.8	27.6 ± 1.5	28.5 ± 1.2	26.5 ± 1.7	21.6 ± 0.9
	LAT	32.8 ± 2.8	25.8 ± 3.5	23.3 ± 3.1	19.1 ± 5.6	16.2 ± 5.0
	SI	32.2 ± 6.4	26.4 ± 5.3	23.8 ± 6.0	21.7 ± 6.1	19.3 ± 5.5
**2.0**	AP	29.5 ± 2.4	27.2 ± 1.9	25.1 ± 2.0	25.6 ± 1.8	26.3 ± 2.9
	LAT	27.7 ± 4.8	24.7 ± 2.9	19.2 ± 4.6	19.0 ± 4.5	18.6 ± 7.0
	SI	26.8 ± 4.5	25.9 ± 5.4	21.9 ± 5.9	21.6 ± 5.0	23.4 ± 7.0

Across all vertex diameters, VPDR values decreased consistently as separation increased, with the smallest diameter (0.5 cm) exhibiting the strongest sensitivity to separation changes (approximately 59-61% decrease from minimum to maximum separation across all three directions). Larger vertex diameters (1.5-2.0 cm) demonstrated enhanced directional uniformity across all separations. Non-coplanar delivery achieved VPDR values below 30% across all three orthogonal directions in 17 out of 20 configurations. The only exceptions were the three configurations with the minimum separation of 1.0 cm for vertex diameters of 0.5, 1.0, and 1.5 cm, where all three directions exceeded 30%. The 2.0 cm diameter achieved VPDR values below 30% across all three directions even at the minimum separation of 1.0 cm.

**Figure 5:**
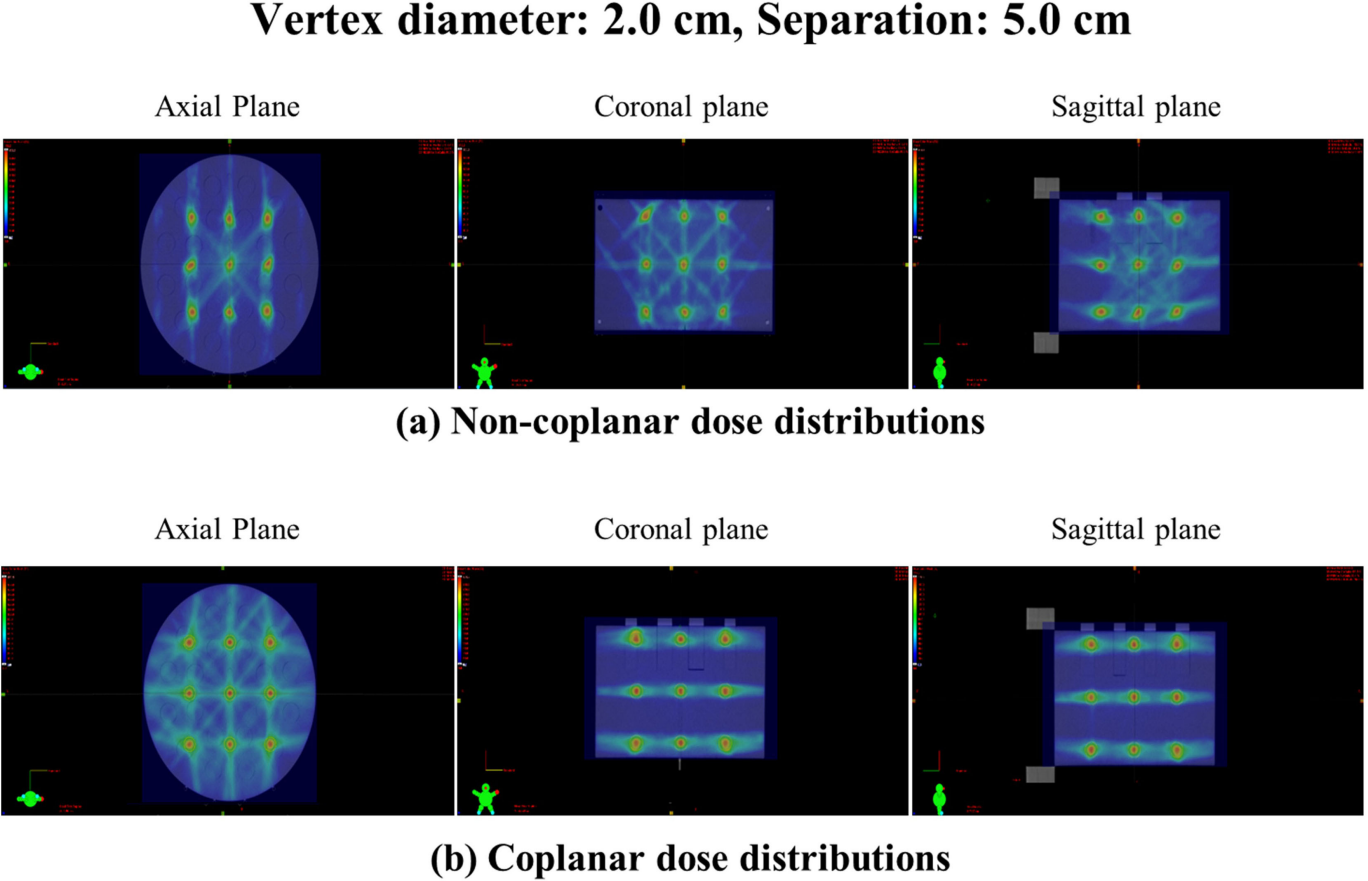
Comparison of dose distributions between non-coplanar and coplanar lattice radiotherapy for vertex diameter 2.0 cm with 5.0 cm separation. **(a)** non-coplanar delivery: dose distributions in axial, coronal, and sagittal planes. **(b)** coplanar delivery: dose distributions in axial, coronal, and sagittal planes [reproduced from Lee et al. (15)].

The three orthogonal directions showed substantially more balanced VPDR distributions compared to coplanar delivery, where SI direction values were consistently 2–3 times lower than transverse directions, as demonstrated by the direct comparison presented in **Table 2**. **Figure 5** presents a direct visual comparison of dose distributions between non-coplanar and coplanar delivery for the representative configuration of 2.0 cm vertex diameter with 5.0 cm separation in axial, coronal, and sagittal planes. The difference in valley dose distribution across the three orthogonal directions between the two delivery approaches is clearly demonstrated. Statistical analysis confirmed that non-coplanar delivery successfully achieved directional VPDR uniformity across all 20 geometric configurations. Pairwise comparisons between directions (AP vs LAT, AP vs SI, LAT vs SI) after Bonferroni correction (α = 0.0008) revealed that directional differences were non-significant in 19/20 configurations for AP vs LAT (p = 0.007–0.939), 20/20 configurations for AP vs SI (p = 0.002–0.752), and 20/20 configurations for LAT vs SI (p = 0.169–0.940). The single significant difference observed (D1.5_S3, AP vs LAT, p = 0.0007) was marginal and did not represent a clinically meaningful directional imbalance given the absolute VPDR difference of less than 5 percentage points. In contrast, coplanar delivery demonstrated statistically significant directional differences in all 20/20 configurations for both AP vs SI (p< 0.0001) and LAT vs SI (p< 0.0001), while AP vs LAT showed no significant difference (0/20 configurations, p = 0.110–0.975), confirming the fundamental directional imbalance inherent to coplanar delivery.

**Table 2:** Comparison of directional VPDR values between coplanar and non-coplanar lattice radiotherapy at 2.0 cm vertex separation.

Diameter	Coplanar (15)			Non-coplanar (this study)		
(cm)	AP (%)	LAT (%)	SI (%)	AP (%)	LAT (%)	SI (%)
0.5	44.8 ± 1.7	44.6 ± 1.9	22.0 ± 3.5	27.8 ± 1.5	25.3 ± 2.9	24.7 ± 4
1.0	38.2 ± 2.8	37.0 ± 2.7	16.9 ± 3.4	24.8 ± 0.5	21.4 ± 3.0	20.2 ± 3.4
1.5	39.7 ± 1.2	40.3 ± 1.1	14.5 ± 0.8	27.6 ± 1.5	25.8 ± 3.5	26.4 ± 5.3
2.0	40.0 ± 1.0	39.8 ± 1.0	11.4 ± 0.7	27.2 ± 1.9	24.7 ± 2.9	25.9 ± 5.4

Coplanar values are from Lee et al. (15). All values are presented as mean ± standard deviation (%). AP, anterior-posterior; LAT, lateral; SI, superior-inferior.

### 3.2 Normal tissue dose and volume analysis

**Figure 6:**
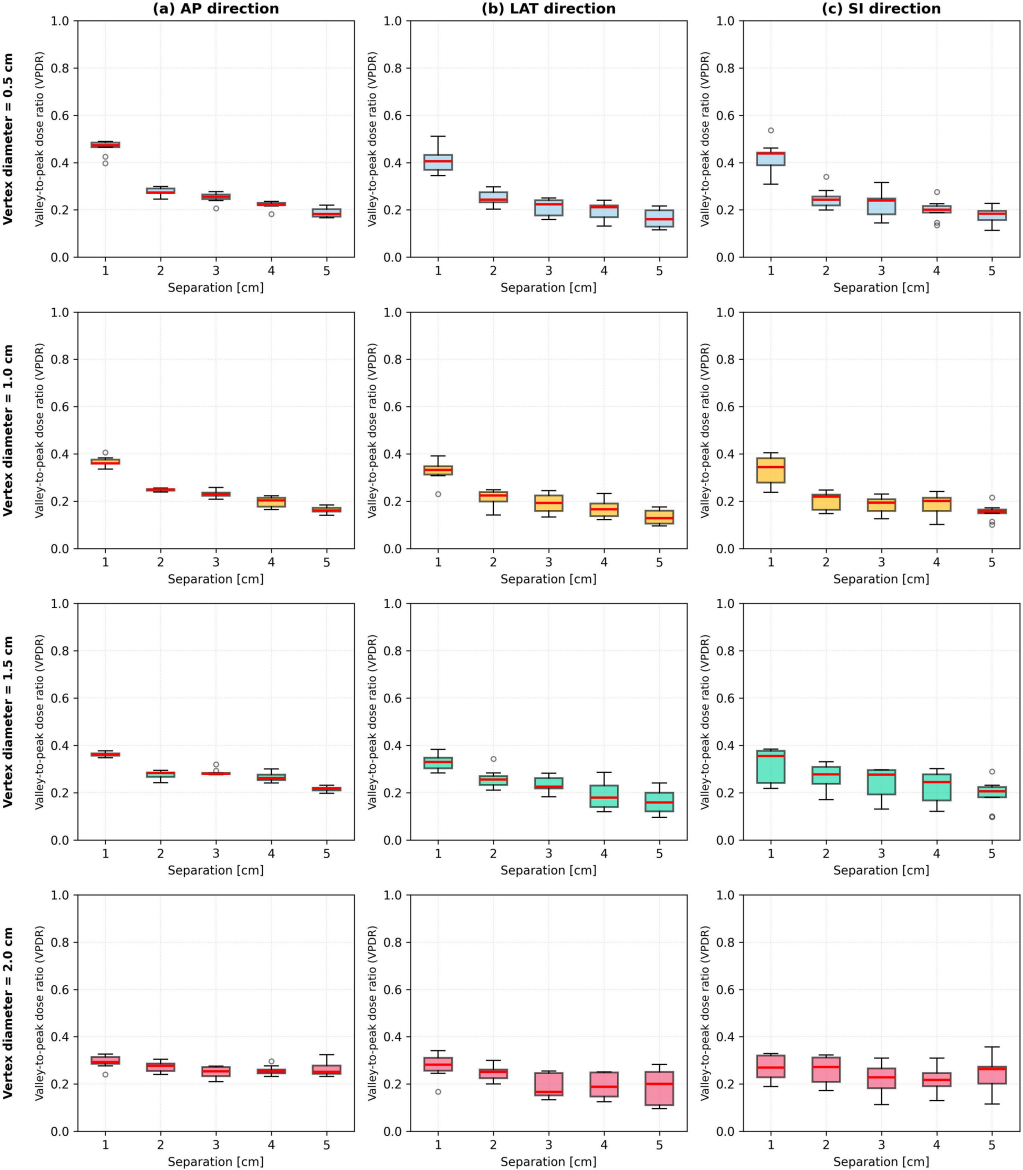
Box plots showing valley-to-peak dose ratio (VPDR) distributions across vertex diameters (0.5, 1.0, 1.5, 2.0 cm) and separations (1–5 cm) in three orthogonal directions: **(a)** anterior-posterior (AP), **(b)** lateral (LAT), and **(c)** superior-inferior (SI). Each box represents the interquartile range with median line, whiskers extend to 1.5× interquartile range, and circles indicate outliers.

Comprehensive VPDR distributions across all vertex diameters and separations are illustrated in **Figure 6**. Normal tissue dosimetric characteristics were evaluated through mean dose and intersecting volume analysis within Eval_Normal_ROI across all geometric configurations. **Table 3** presents detailed results for each vertex diameter and separation combination.

**Table 3:** Evaluation of normal tissue dose parameters based on vertex configurations in non-coplanar LRT.

Diameter (cm)	Evaluation items	Separation (cm)				
		1	2	3	4	5
0.5	Mean dose (cGy)	59.2	95.2	123.2	142.2	165.9
	Intersecting volume (cm^3^)	0.3	0.2	0.0	0.0	0.0
1	Mean dose (cGy)	99.2	145.3	167.2	193.9	209.6
	Intersecting volume (cm^3^)	1.7	0.0	0.0	0.0	0.0
1.5	Mean dose (cGy)	157.4	207.2	238.6	245.1	252.7
	Intersecting volume (cm^3^)	10.1	1.2	0.5	0.1	0.0
2	Mean dose (cGy)	200.4	233.8	266.1	280.4	310.4
	Intersecting volume (cm^3^)	63.1	30.2	28.8	14.2	10.3

#### 3.2.1 Mean dose to normal tissue

Mean dose to Eval_Normal_ROI demonstrated systematic increases with both vertex diameter and separation across all configurations (**Table 3**). Larger vertex diameters consistently delivered higher integral doses to surrounding normal tissue, with mean dose ranging from 59.2-165.9 cGy for 0.5 cm diameter to 200.4-310.4 cGy for 2.0 cm diameter across all separations. The rate of increase with separation was inversely related to vertex diameter, with smaller vertices showing greater sensitivity to separation changes (2.8-fold increase for 0.5 cm vs. 1.5-fold increase for 2.0 cm diameter from minimum to maximum separation).

#### 3.2.2 Intersecting volume analysis

Intersecting volume, defined as Eval_Normal_ROI volume receiving ≥50% prescription dose (10.0 Gy), exhibited strong dependence on vertex diameter and separation (**Table 3**). Smaller vertex diameters achieved effective isolation of high-dose regions from normal tissue at relatively modest separations: zero intersecting volume was achieved at separations ≥3.0 cm for 0.5 cm diameter and ≥2.0 cm for 1.0 cm diameter. Larger vertex diameters required greater separations for adequate normal tissue protection, with the 1.5 cm diameter reaching zero intersecting volume only at 5.0 cm separation. In contrast, the 2.0 cm diameter demonstrated substantial intersecting volumes across all evaluated separations (10.3 cm³ even at 5.0 cm separation), indicating that large vertices require particularly careful consideration of separation distance in clinical implementation.

### 3.3 Monitor unit distribution and plan complexity analysis

Treatment delivery characteristics were analyzed through total MU requirements, arc-specific MU contributions, and modulation complexity scores across all geometric configurations.

#### 3.3.1 Total monitor unit requirements

**Figure 7:**
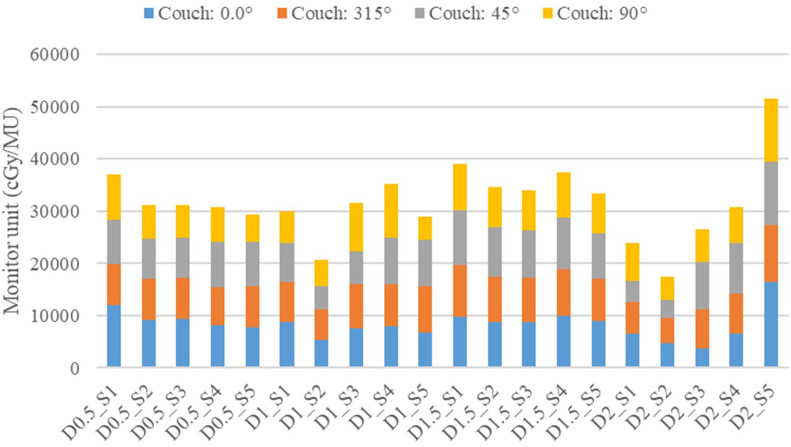
Monitor unit (MU) distribution across different couch angles for all 20 geometric configurations. Stacked bar chart shows the contribution of each couch angle (0°: blue, 315°: orange, 45°: gray, 90°: yellow) to total MU delivery, with total MU values ranging from 17,524 to 51,488 depending on vertex geometry. Configuration labels indicate diameter (D) and separation (S) in cm (e.g., D0.5_S1 = 0.5 cm diameter with 1.0 cm separation).

Total MU values for non-coplanar plans varied between 17,524 MU and 51,488 MU across all configurations (**Table 4**), reflecting the diverse optimization requirements for different vertex geometries. **Figure 7** illustrates the variation in total MU and individual arc contributions according to vertex diameter and separation. No consistent pattern emerged correlating total MU with either diameter or separation independently, suggesting that HyperArc optimization adapts MU requirements based on the combined effects of vertex geometry and spatial configuration rather than individual parameters.

**Table 4:** Variation in total MU and MCS with changes in diameter and separation.

Diameter (cm)	Separation (cm)	Total MU	Couch: 0.0° gantry: 180.1°-179.9°		Couch: 315.0° gantry: 179.9°-0.0°		Couch: 45.0° gantry: 0.0°-180.1°		Couch: 90.0° gantry: 180.1°-0.0°		MCS
			MU	%	MU	%	MU	%	MU	%	
0.5	**1**	37126.2	12045.3	32.4	7913.3	21.3	8308.3	22.4	8859.3	23.9	0.34
	**2**	31164.0	9119.3	29.3	7842.3	25.2	7758.9	24.9	6443.5	20.7	0.28
	**3**	31162.9	9406.5	30.2	7829.2	25.1	7594.7	24.4	6332.5	20.3	0.29
	**4**	30853.6	8240.3	26.7	7284.0	23.6	8651.6	28.0	6677.7	21.6	0.28
	**5**	29464.0	7776.1	26.4	7832.7	26.6	8518.2	28.9	5337.0	18.1	0.36
1.0	**1**	29968.4	8667.5	28.9	7752.3	25.9	7590.7	25.3	5957.9	19.9	0.36
	**2**	20766.4	5279.4	25.4	5989.5	28.8	4458.5	21.5	5039.0	24.3	0.40
	**3**	31644.8	7646.5	24.2	8295.6	26.2	6256.5	19.8	9446.2	29.9	0.36
	**4**	35186.6	7929.1	22.5	8003.3	22.7	8935.5	25.4	10318.7	29.3	0.36
	**5**	29026.1	6682.7	23.0	8925.6	30.8	8826.3	30.4	4591.5	15.8	0.40
1.5	**1**	39111.4	9871.9	25.2	9816.0	25.1	10551.2	27.0	8872.3	22.7	0.47
	**2**	34690.2	8790.4	25.3	8658.9	25.0	9426.5	27.2	7814.4	22.5	0.47
	**3**	34012.1	8786.4	25.8	8437.8	24.8	9028.6	26.5	7759.3	22.8	0.33
	**4**	37450.4	9878.6	26.4	9012.5	24.1	9909.7	26.5	8649.6	23.1	0.32
	**5**	33377.0	8953.7	26.8	8100.5	24.3	8601.2	25.8	7721.6	23.1	0.38
2.0	**1**	23992.3	6549.6	27.3	6050.4	25.2	4097.8	17.1	7294.5	30.4	0.44
	**2**	17524.1	4710.6	26.9	4822.8	27.5	3524.0	20.1	4466.7	25.5	0.41
	**3**	26513.2	3695.3	13.9	7395.5	27.9	9096.1	34.3	6326.3	23.9	0.31
	**4**	30830.6	6562.6	21.3	7572.1	24.6	9816.8	31.8	6879.1	22.3	0.33
	**5**	51487.8	16426.3	31.9	10983.4	21.3	12120.4	23.5	11957.7	23.2	0.32

#### 3.3.2 Arc-specific monitor unit distribution

Analysis of MU distribution across the four non-coplanar arcs (couch 0°, 315°, 45°, 90°) revealed generally balanced contribution patterns, with individual arc contributions ranging from 13.9% to 34.3% across all configurations, and 82.5% of all arc contributions falling within the 20-30% range (**Table 4**). This relatively uniform distribution across couch angles contrasts markedly with coplanar delivery, where specific collimator angles (315° and 45°) dominated MU contribution (>80% in coplanar plans (15)), indicating more effective utilization of non-coplanar geometry for achieving directionally uniform dose delivery.

#### 3.3.3 Plan complexity analysis

Modulation complexity scores ranged from 0.28 to 0.47 across all configurations (**Table 4**), indicating moderate plan complexity comparable to values reported for advanced IMRT and VMAT techniques. The smallest vertex diameter (0.5 cm) generally showed lower MCS values (0.28-0.36) compared to larger diameters (1.0-2.0 cm: 0.31-0.47), reflecting the increased modulation required to achieve steep dose gradients between closely spaced small vertices.

### 3.4 Representative patient-based planning case

**Figure 8:**
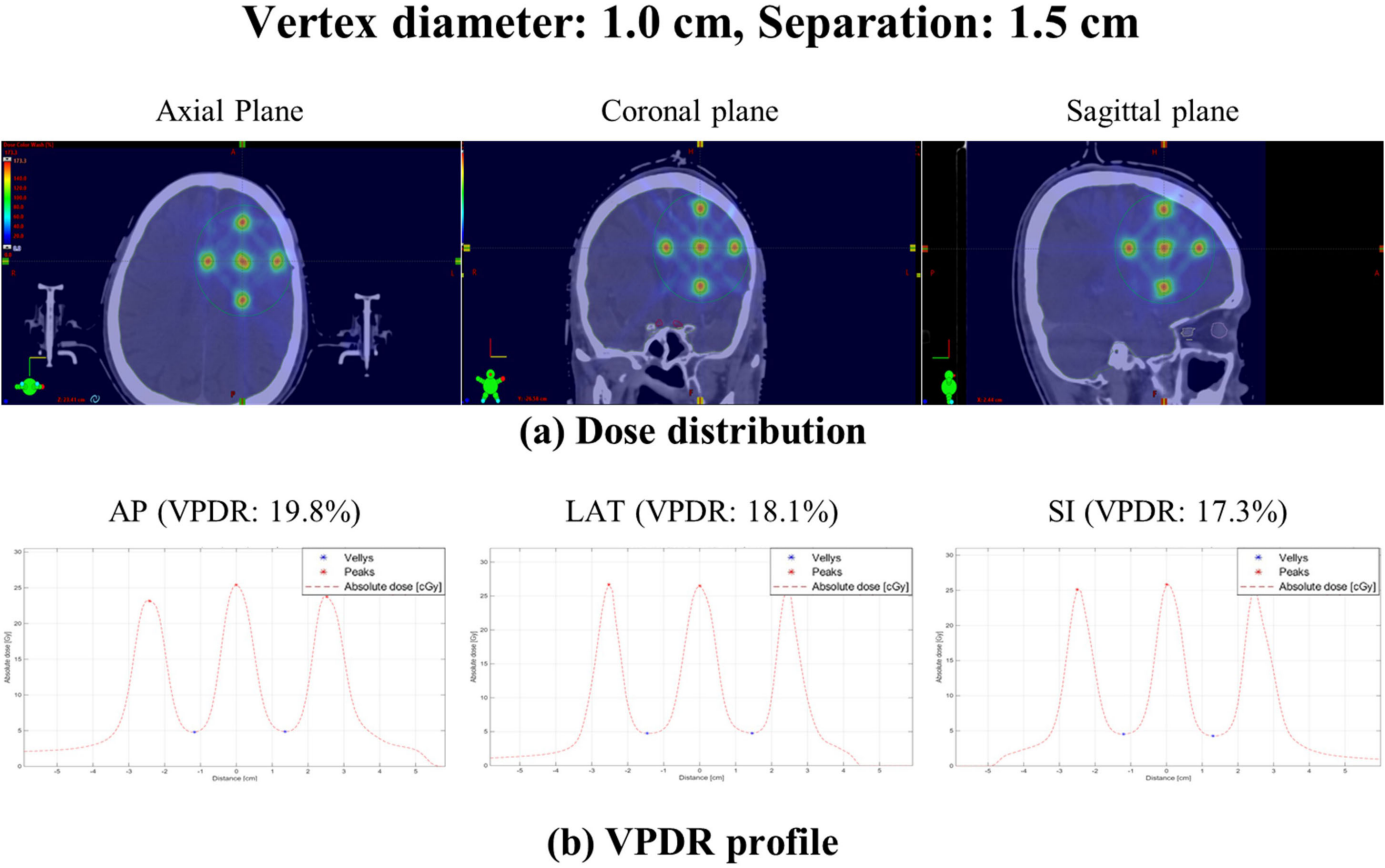
Representative patient-based planning case demonstrating the clinical applicability of non-coplanar lattice radiotherapy for a brain tumor with a gross tumor volume of 173.8 cm³. Vertex diameter of 1.0 cm and edge-to-edge separation of 1.5 cm were applied, with 7 vertices placed within the target volume. **(a)** Dose distributions in axial, coronal, and sagittal planes. **(b)** VPDR profiles in AP (19.8%), LAT (18.1%), and SI (17.3%) directions showing peak and valley dose patterns. Peak doses represent the maximum dose within each vertex volume and valley doses represent the minimum dose within the region between two consecutive vertices. Both values were identified using MATLAB with inter-point distance normalization.

To demonstrate the clinical applicability of the proposed non-coplanar LRT approach, a representative patient-based planning case was performed using a brain tumor patient CT. The cranial anatomy permits couch rotation without collision risk, representing a clinically relevant site for non-coplanar LRT, and recent investigations have demonstrated the feasibility of LRT for intracranial applications (24). A spherical gross tumor volume of 173.8 cm³ was contoured, and non-coplanar LRT was planned with a vertex diameter of 1.0 cm and an edge-to-edge separation of 1.5 cm. Due to the tumor volume constraints, 7 vertices were placed within the target volume, compared to the 27-vertex arrangement used in the phantom study. **Figure 8** presents the dose distributions in axial, coronal, and sagittal planes, along with VPDR profiles in three orthogonal directions. The resulting VPDR values were 19.8% (AP), 18.1% (LAT), and 17.3% (SI), demonstrating directionally balanced dose distribution across all three orthogonal directions, consistent with the dosimetric trends observed in the phantom study.

## 4 Discussion

This study evaluated the technical feasibility of non-coplanar lattice radiotherapy and systematically analyzed its advantages in achieving directional VPDR uniformity compared to coplanar delivery. The findings demonstrate that non-coplanar beam arrangements utilizing various couch angles (0°, 315°, 45°, 90°) successfully address the directional VPDR imbalance observed in our previous coplanar investigation (15), achieving substantially more uniform three-dimensional dose distribution across comprehensive vertex parameter combinations.

### 4.1 Quantitative comparison with coplanar delivery

Direct comparison with our previous coplanar study (15) reveals the magnitude of change achieved through non-coplanar delivery. **Table 2** presents matched configurations demonstrating the redistribution of VPDR values across directions. For the representative configuration of 1.5 cm vertex diameter with 2.0 cm separation, non-coplanar delivery achieved SI direction VPDR of 26.4 ± 5.3%, representing an 82% increase from the coplanar value of 14.5 ± 0.8%. Simultaneously, AP and LAT directions showed moderate reductions from 39.7 ± 1.2% and 40.3 ± 1.1% (coplanar) to 27.6 ± 1.5% and 25.8 ± 3.5% (non-coplanar), respectively.

The redistribution pattern was consistent across the entire parameter space. For 1.0 cm diameter with 2.0 cm separation, SI VPDR increased from 16.9 ± 3.4% (coplanar) to 20.2 ± 3.4% (non-coplanar), a 19.5% increase, while AP and LAT values decreased from 38.2 ± 2.8% and 37.0 ± 2.7% to 24.8 ± 0.5% and 21.4 ± 3.0%, respectively. Similarly, for 2.0 cm diameter with 2.0 cm separation, SI VPDR increased from 11.4 ± 0.7% to 25.9 ± 5.4% (a 127% increase), while transverse directions decreased from approximately 40% to 27%.

These data demonstrate that non-coplanar delivery consistently redistributes VPDR values across directions, substantially reducing the directional imbalance observed in coplanar delivery. In the representative configuration of 1.5 cm diameter with 2.0 cm separation, the 2.7-fold difference between SI and transverse VPDR values observed in coplanar delivery was resolved, with all three directions achieving comparable VPDR values in non-coplanar delivery. Most importantly, this redistribution resolves the fundamental directional imbalance inherent to coplanar delivery, where a 2.7-fold difference between SI and transverse VPDR values created highly non-uniform spatial fractionation across three dimensions. Non-coplanar delivery successfully equalizes VPDR across all three orthogonal directions, bringing them within the dosimetric range commonly reported in prior LRT studies (6). It should be noted that this directional imbalance was observed under the specific conditions of this phantom study, utilizing a uniformly distributed 3×3×3 vertex arrangement with fixed center-to-center spacing. In clinical practice, patient-specific adaptation of vertex geometry, such as reducing center-to-center distance in the SI direction, may partially mitigate the directional imbalance observed with coplanar delivery. Nevertheless, within the systematic parameter space evaluated in this study, coplanar delivery consistently demonstrated pronounced directional imbalance, with transverse directions showing VPDR values of approximately 40% while the SI direction fell below 20% for most configurations (15).

The observed redistribution can be directly attributed to the geometric principles discussed in the introduction. In coplanar delivery, beam trajectories confined to a single axial plane create natural pathways connecting vertices in AP and LAT directions but not in the SI direction, resulting in higher valley doses (higher VPDR) in transverse directions and lower valley doses (lower VPDR) along the cranial-caudal axis. Non-coplanar delivery introduces beam trajectories with SI directional components through couch rotation, providing radiation pathways that connect cranial-caudally separated vertices. This geometric modification directly increases valley doses in the SI direction while distributing dose more uniformly in three dimensions, thereby balancing VPDR values across all directions.

### 4.2 Clinical implications and parameter selection

The achievement of balanced directional VPDR distribution has potential clinical implications for LRT implementation. From a radiobiological perspective, the spatial pattern of low-dose valleys influences normal tissue recovery and immune activation. While the coplanar approach creates concentrated low-dose corridors primarily in the SI direction with excessive doses in transverse directions, the non-coplanar approach distributes valleys more uniformly throughout the three-dimensional space while achieving substantially more balanced directional VPDR distribution. This difference may affect normal tissue recovery patterns, as the dose-volume effect and vascular preservation mechanisms depend on the spatial configuration of spared tissue regions (14, 16). Additionally, recent evidence suggests that the spatial pattern of dose heterogeneity influences immune response activation (16, 17), though the optimal geometric configuration for maximizing immunological outcomes remains an area requiring biological investigation.

For clinical implementation, parameter selection must balance multiple considerations. Our results demonstrate that achieving directionally balanced VPDR values requires careful attention to both vertex geometry and delivery approach. While lower VPDR values indicate stronger spatial dose modulation within the tumor, the more critical finding of this study is that directional uniformity — rather than absolute VPDR values alone — represents an important dosimetric consideration for LRT implementation. The 2.7-fold directional imbalance observed in coplanar delivery implies that spatial fractionation effects may vary substantially depending on the anatomical direction, potentially compromising the biological rationale of three-dimensional LRT. Non-coplanar delivery addresses this limitation by distributing VPDR values uniformly across all three orthogonal directions within the dosimetric range commonly reported in prior LRT studies (6). For non-coplanar delivery, configurations achieving near-optimal VPDR balance include: 1.5 cm diameter with 2.0-3.0 cm separation (VPDR range: 23.3-28.5% across directions); 2.0 cm diameter with 2.0-3.0 cm separation (VPDR range: 19.2-27.2%); and 1.0 cm diameter with 3.0-4.0 cm separation (VPDR range: 17.2-23.1%). These configurations achieve substantially reduced directional VPDR coefficients of variation compared to coplanar delivery (3.4–13.4% for recommended configurations, vs. 50–60%), indicating improved directional uniformity with non-coplanar geometry.

The choice between coplanar and non-coplanar delivery involves trade-offs. Non-coplanar delivery provides superior directional uniformity but requires additional couch rotations, increasing treatment time and potentially introducing small positioning uncertainties despite modern 6-degree-of-freedom couch capabilities (10). Our MU analysis reveals that non-coplanar delivery shows balanced beam weight distribution across different couch angles, with no single angle dominating, suggesting efficient dose delivery. The MCS values ranging from 0.28 to 0.47 across configurations (**Table 4**) indicate moderate plan complexity. While these values suggest more complex delivery requirements than simple field arrangements, they remain well within deliverable ranges for modern linear accelerators equipped with high-resolution MLC systems. The observed MCS range of 0.28-0.47 is consistent with values reported for other complex VMAT plans, including head and neck (0.22), pelvic (0.36), and thoracic (0.19) treatments (25), as well as various clinical VMAT sites with MCS values ranging from 0.19 to 0.65 (26), confirming technical feasibility for clinical delivery. No consistent correlation between MCS and either vertex diameter or separation was observed, suggesting that plan complexity in non-coplanar LRT is determined by the combined interaction of vertex geometry and spatial configuration rather than by individual parameters alone.

### 4.3 Comparison with previous non-coplanar SFRT studies

While several studies have reported clinical implementation of LRT (9, 10, 18, 21), systematic dosimetric evaluation of non-coplanar delivery has been limited. Gaudreault et al. reported automated planning approaches for LRT but focused on coplanar delivery optimization (27). Ertan et al. compared MLC-based and cone-based SFRT delivery but did not systematically evaluate directional VPDR patterns (28). Our study uniquely provides comprehensive quantitative comparison between coplanar and non-coplanar approaches across a wide parameter space using identical methodology, enabling direct assessment of the geometric modification’s impact on dose distribution.

Direct quantitative comparison with previous LRT studies is limited by differences in delivery technique, vertex geometry, and dosimetric methodology. Nevertheless, the directional VPDR imbalance observed in our previous coplanar study (15) has been independently corroborated by Seol et al. using helical tomotherapy-based LRT (29), where SI direction consistently showed lower VPDR values than transverse directions. The present study is the first to systematically demonstrate that non-coplanar beam delivery can resolve this directional imbalance across a comprehensive parameter space.

### 4.4 Normal tissue considerations

Normal tissue dose analysis revealed important trends. Mean dose to Eval_Normal_ROI increased with both vertex diameter and separation, ranging from 59.2 cGy (0.5 cm diameter, 1.0 cm separation) to 310.4 cGy (2.0 cm diameter, 5.0 cm separation). These values represent relatively low integral dose to normal tissues, particularly for small vertex configurations. The intersecting volume analysis—measuring normal tissue receiving ≥50% prescription dose—demonstrated that 1.0 cm diameter achieved zero intersection at separations ≥2.0 cm, while 0.5 cm diameter achieved zero intersection at separations ≥3.0 cm, indicating effective normal tissue protection from high-dose regions. However, larger diameters showed persistent intersection even at maximum separations, with 2.0 cm diameter maintaining 10.3 cm³ intersection at 5.0 cm separation.

These findings suggest that small- to medium-sized vertex configurations (0.5-1.5 cm) with adequate separation (≥2.0 cm) provide optimal balance between achieving therapeutic VPDR and minimizing high-dose exposure to normal tissues.

### 4.5 Technical considerations and future directions

Several technical aspects merit consideration for clinical implementation. The consistent contribution of collimator angles 315° and 45° to MU distribution (typically 60-85% combined) observed in our study, which persisted across coplanar (15) and non-coplanar configurations, suggests these angles are particularly effective for creating SFRT dose distributions with the Millennium 120 MLC. This pattern may inform beam angle optimization strategies, though the optimal configuration may vary with tumor location and anatomy.

The observation that 2.0 cm diameter maintained relatively stable VPDR across separation changes, while smaller diameters showed high sensitivity to separation, relates to the physical characteristics of the Millennium 120 MLC. The 2.0 cm diameter, being four times the central leaf width (5 mm), allows stable beam formation, whereas smaller diameters approach the leaf width resolution limit, increasing geometric uncertainty. This technical constraint should inform parameter selection for clinical implementation. Furthermore, it should be noted that the dosimetric outcomes reported in this study are specific to the Eclipse HyperArc optimization algorithm. Different treatment planning systems or optimization algorithms may generate different MLC segmentation patterns and beam configurations, potentially leading to different VPDR distributions even for identical vertex geometries. Therefore, the parameter recommendations derived from this study should be validated when implemented with alternative TPS platforms.

The total MU values observed in this study reflect a known characteristic of HyperArc-based automated optimization, which typically requires higher MU compared to conventional VMAT planning due to increased modulation complexity associated with simultaneous non-coplanar arc optimization. Tamura et al. reported that HyperArc VMAT required significantly higher MU than conventional VMAT (8,186 ± 1,390 MU vs. 6,758 ± 1,450 MU, p< 0.01), with correspondingly lower modulation complexity scores (30).

From a safety perspective, the non-coplanar couch angles employed in this study (45°, 315°, and 90°) may pose significant gantry-patient collision risks in clinical practice, and mandatory collision checks with individualized beam arrangement optimization are strongly recommended prior to clinical implementation, as detailed in the Limitations section.

Future investigations should address several important areas. First, patient-specific studies across various tumor sites and anatomies are needed to validate these phantom-based findings and establish site-specific parameter recommendations. Respiratory motion effects, particularly relevant for thoracic and abdominal sites, require dedicated evaluation as vertex position uncertainties could affect VPDR achievement. Second, biological validation studies correlating directional VPDR patterns with normal tissue toxicity and tumor control outcomes would provide critical evidence for the clinical relevance of directional uniformity. Third, optimization algorithms specifically designed for non-coplanar LRT could potentially improve upon the HyperArc-based approach used in this study, perhaps achieving target VPDR ranges with improved efficiency. Finally, integration with emerging technologies such as real-time imaging and adaptive planning could enhance the precision and reliability of LRT delivery.

### 4.6 Limitations

This study has several limitations that should be acknowledged. First, as a phantom-based investigation, the findings do not account for patient-specific anatomical variations, tissue heterogeneities, or organ motion that would be present in clinical settings. The cylindrical Virtual Water phantom provides ideal conditions for systematic parameter evaluation but does not reflect the complexity of actual patient anatomy. Second, we evaluated a fixed lattice structure (3×3×3, 27 vertices) to enable systematic comparison with our previous coplanar study (15). However, fixing the number of vertices while varying diameter and separation results in changes to the proportion of tumor volume occupied by lattice vertices across configurations, which may influence the interpretation of normal tissue dose characteristics. Future investigations should evaluate the impact of varying vertex number alongside diameter and separation to provide more comprehensive clinical guidance. Third, the study did not include experimental validation through phantom measurements; while the AAA dose calculation algorithm is well-validated for conventional fields, independent verification of calculated VPDR values through measurement would strengthen confidence in the results. Fourth, we did not evaluate the impact of different beam energies or dose rates, which may influence achievable VPDR distributions. Fifth, the study focused on dosimetric characterization without biological endpoint evaluation; the clinical significance of improved directional VPDR uniformity requires validation through biological studies and clinical trials. Additionally, although the non-coplanar beam configurations evaluated in this phantom study demonstrated favorable dosimetric characteristics, the couch angles employed (45°, 315°, and 90°) may pose significant gantry-patient collision risks in actual clinical scenarios. In single-fraction LRT, maximizing the arc range (200°–360°) is strongly recommended to ensure adequate dose delivery, which inherently restricts the feasibility of large couch rotations. Furthermore, when non-coplanar LRT is combined with advanced techniques such as surface-guided radiation therapy (SGRT) or deep inspiration breath hold (DIBH), the associated immobilization devices — including camera systems, masks, and vacuum cushions — may further limit the available range of couch rotation due to elevated collision probability. Therefore, prior to clinical implementation, mandatory collision checks and individualized beam arrangement optimization based on patient-specific anatomy and immobilization setup are strongly recommended. Furthermore, all treatment plans in this study utilized a fixed collimator angle of 45° across all four non-coplanar arcs. This angle was adopted based on the HyperArc algorithm recommendation and was intentionally held constant across all 20 geometric configurations to isolate the effects of vertex diameter and separation on directional VPDR distribution, thereby preventing collimator angle from acting as a confounding variable in the systematic evaluation. However, collimator angle is a key parameter that influences MLC segmentation, monitor unit requirements, and overall plan complexity in a treatment planning system-dependent manner. The use of a fixed collimator angle may not represent the optimal configuration for each individual vertex geometry, and systematic optimization of collimator angle for each specific configuration could potentially yield different VPDR distributions and plan complexity metrics. Future investigations should evaluate the impact of collimator angle optimization on directional VPDR uniformity in non-coplanar LRT delivery. Finally, we evaluated a specific MLC system (Millennium 120); results may differ for other MLC designs with different leaf widths or positioning characteristics.

## 5 Conclusion

This study demonstrates that non-coplanar lattice radiotherapy using the Millennium 120 MLC successfully achieves balanced directional VPDR distribution, addressing the directional imbalance inherent in coplanar delivery. Through systematic evaluation across comprehensive vertex parameter combinations, we established that non-coplanar delivery redistributes VPDR values across directions, with SI direction values increasing by 19-127% depending on configuration while moderately reducing transverse direction values. This redistribution results in three-dimensional uniformity improvement. Directional coefficient of variation was substantially reduced from 50-60% (coplanar) to 3.4-17.1% across all configurations (non-coplanar). Most importantly, non-coplanar delivery enables achieving directionally balanced VPDR values below 30% across all three orthogonal directions simultaneously with appropriate parameter selection, which was not achievable with our previous coplanar delivery (15), where transverse directions consistently exceeded 30% while SI direction fell below 20% for most configurations. These findings provide quantitative evidence supporting non-coplanar LRT as a technically feasible approach offering improved dosimetric characteristics compared to coplanar delivery, with practical guidance for parameter selection to achieve directionally balanced VPDR distributions while maintaining normal tissue protection. Future patient-specific studies and biological validation are needed to establish the clinical significance of these dosimetric improvements.

## Data Availability

The original contributions presented in the study are included in the article/supplementary material. Further inquiries can be directed to the corresponding authors.
